# Protective effects of tanshinone Ⅰ against cisplatin-induced nephrotoxicity in mice

**DOI:** 10.22038/IJBMS.2022.58959.13102

**Published:** 2022-03

**Authors:** Yan Wang, Yun-hui Zhang, Yin-ru Tang, Jie Lan, Zhi-ying Huang, Wei Tian, Qian Huang, Yan Peng, Yuan Gao, Yue-qin Hu, Xue-nong Zhang

**Affiliations:** 1 Department of Pharmacy, The First College of Clinical Medical Science, China Three Gorges University & Yichang Central People’s Hospital, Yichang 443003, P.R. China; 2 School of Pharmaceutical Sciences, Sun Yat-sen University, Guangzhou 510006, P.R. China; 3 Department of Nephrology, The First College of Clinical Medical Science, China Three Gorges University & Yichang Central People’s Hospital,Yichang 443003, P.R. China

**Keywords:** Cisplatin, Mice, Nephrotoxicity, Oxidative stress, Tanshinone Ⅰ

## Abstract

**Objective(s):**

Cisplatin (CDDP) is a highly effective chemotherapeutic agent, but its clinical application has been limited by nephrotoxicity. Tanshinone Ⅰ (T-I), a phenanthrenequinone compound extracted from the Chinese herb Danshen, has been used to improve circulation and treat cardiovascular diseases. The aim of this study was to investigate the protective effect of T-I on CDDP-induced nephrotoxicity in mice.

**Materials and Methods:**

The BALB/c mouse models of nephrotoxicity were established by a single intraperitoneal injection of 20 mg/kg CDDP on the first day of the experiment. Three hours prior to CDDP administration, the mice were dosed with 10 mg/kg and 30 mg/kg T-I for 3 consecutive days intraperitoneally to explore nephroprotection of T-I.

**Results:**

Treatment with T-I significantly reduced blood urea nitrogen and creatinine levels in serum observed in CDDP-administered mice, especially at a dose of 30 mg/kg. T-I at 30 mg/kg significantly decreased malondialdehyde levels and increased glutathione levels and the enzymatic activity of catalase in kidney tissue compared to CDDP. Additionally, T-I (30 mg/kg) significantly reversed the CDDP-decreased expression level of superoxide dismutase 2 protein in renal tissue. Histopathological evaluation of the kidneys further confirmed the protective effect of T-I.

**Conclusion:**

The findings of this study demonstrate that T-I can protect against CDDP-induced nephrotoxicity through suppression of oxidative stress.

## Introduction

Cisplatin (*cis*-diamminedichloroplatinum Ⅱ, CDDP), as a platinum-containing drug, is widely used in clinical practice for a variety of cancers, including bladder, ovarian, lung, and testicular cancer (1). However, it is limited by severe adverse side effects, such as nephrotoxicity, gastrointestinal reaction, myelosuppression, neurotoxicity and ototoxicity, particularly nephrotoxicity, which is the main dose-limiting factor (2). It has been reported that 20-30% of patients treated with CDDP developed evidence of acute kidney injury (3). CDDP accumulates mainly in the kidney, and the mechanisms of its nephrotoxicity include DNA damage, cytoplasmic organelle dysfunction, apoptosis, inflammation and oxidative stress (3, 4). Oxidative stress induced by the accumulation of intracellular reactive oxygen species (ROS) is a hallmark of CDDP-induced nephrotoxicity, which is related to glutathione (GSH) depletion, a decrease in anti-oxidant enzyme activity, and an increase in lipid peroxidation and oxygen free radicals in the kidney (1, 5). If ROS are not properly neutralized, they will lead to the dysregulation of some signaling pathways, such as MAPK, PI3K, Nrf2, iron metabolism, DNA damage response, and cell death (6). Recently, several studies have demonstrated that inhibition of oxidative stress can reduce CDDP-induced nephrotoxicity **(**5, 7).

Tanshinone Ⅰ (T-I) is one of the major phenanthrenequinone compounds extracted from the Chinese herb Danshen (*Salvia miltiorrhiza *Βunge), which has been used to improve circulation and treat cardiovascular diseases in China (8). Recent studies have shown that T-I can enhance anti-oxidative activity against pro-oxidant challenge, thereby presenting potential neuroprotection against neuronal damage and peroxynitrite-induced DNA damage (8, 9). Furthermore, T-I has been proven to be a NF-E2 p45-related factor 2 (Nrf2)-activator that can activate the Nrf2-dependent anti-oxidant response and protect against arsenic (As) (III)-induced lung inflammation *in vitro* and *in vivo *(10). In addition, a previous study showed that T-I could facilitate the metabolism of aristolochic acid I (AAI) and prevent AAI-induced kidney injury by inducing hepatic CYP1A 1/2 in mice (11).

 A previous study in our laboratory had demonstrated that T-I can increase cell viability, suppress the increased intracellular ROS, and activate the Nrf2 signaling pathway to attenuate CDDP-induced cytotoxicity in HK-2 cells (human proximal tubular epithelial cell line)(12). Building on this foundation, we continue to investigate the protective effect of treatment with T-I on CDDP-induced nephrotoxicity in mice.

## Materials and Methods

***Chemicals and kits***

Tanshinone I (>97% purity) was purchased from Shanghai Macklin Biochemical Co, Ltd (Shanghai, China). CDDP (>65% purity) and corn oil were purchased from Shanghai Aladdin Biochemical Tech Co, Ltd (Shanghai, China). Reduced glutathione (GSH), malondialdehyde (MDA) and catalase (CAT) assay kits were purchased from Nanjing Jiancheng Biotech Co, Ltd (Nanjing, China). The Pierce™ BCA protein assay kit was purchased from Thermo Fisher Scientific Inc. (MA, USA). Anti-superoxide dismutase 2 (SOD2) antibody and anti-GAPDH antibody were purchased from Cell Signaling Technology (Beverly, MA, USA). Other chemicals were of analytical grade from commercial suppliers.

***Animals***

 Specific pathogen-free (SPF) male BALB/c mice (18-22 g) were purchased from the Laboratory Animal Center of Sun Yat-sen University. All mice were housed under standard SPF conditions controlled at a temperature of 20~25 °C and humidity of 40~70%, with a 12-hour light-dark cycle. Food and water were provided ad libitum. The animal study was approved by the Animal Ethics and Welfare Committee of Sun Yat-sen University (Approval No.: SYSU-IACUC-2019-000337).

***Experimental protocols***

**Figure 1:**
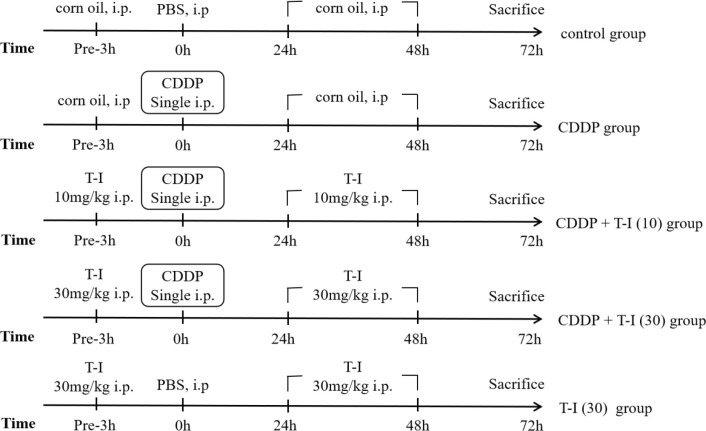
Animal treatment protocols. Twenty-five mice were randomly assigned into five groups: control, CDDP, CDDP + T-I (10), CDDP + T-I (30) and T-I (30) (each group, five mice). CDDP: cisplatin; T-I: tanshinone I; PBS: phosphate buffered saline

Mice were randomly assigned into five groups (n=5). Group I (control group) were intraperitoneally (IP) treated with corn oil (vehicle of T-I) and PBS (vehicle of CDDP), as shown in Figure 1. Group II (CDDP group) were administered a single dose of CDDP (20 mg/kg (13), IP) after 3 hr of corn oil treatment, and then corn oil was given for 2 consecutive days. Group III (CDDP + T-I 10 group) and Group IV (CDDP + T-I 30 group) were firstly treated with T-I (10, 30 mg/kg, IP) and then given a single dose of CDDP (20 mg/kg, IP) 3 hr later, and subsequently, they received T-I (10, 30 mg/kg, IP) for 2 consecutive days(11, 14). Group V (T-I 30 group) were treated with T-I (30mg/kg, IP) 3 hr prior to PBS treatment on the first day, and then given T-I (30 mg/kg, IP) once a day for the next 2 days. The mice were euthanized 72 hr after CDDP treatment (Figure 1). Blood samples were collected to evaluate serum creatinine (CRE) and blood urea nitrogen (BUN) levels. Kidneys were removed and weighed immediately. The left kidney was fixed in 10% formalin for histopathological studies, while the other kidney was stored at -80℃ for subsequent detection.

***Assessment of renal function and oxidative stress***

The levels of serum CRE and BUN were measured by a CX5 automatic analyzer (Beckman, USA) using standardized commercially available kits (Leadman Biochemistry Co. Beijing, China). Kidneys were homogenized with saline at a weight-to-volume ratio of 1:9. GSH, MDA and CAT assays were performed according to the manufacturers’ instructions. The protein concentration was determined using the BCA Protein Assay Kit (Thermo Fisher Scientific).

***Western blot analysis***

Total protein was extracted from the kidney tissues using cell lysis buffer for Western and IP (Beyotime Institute of Biotechnology, Shanghai, China). Extracts were separated by 10% SDS-polyacrylamide gels electrophoretically and then transferred to polyvinylidene difluoride (PVDF) membranes (Millipore Co, Billerica, MA, USA). After being blocked in 5% nonfat milk in TBST for 1 hr at room temperature, the membranes were incubated with primary antibodies at 4℃ overnight, washed and conjugated with secondary antibodies at room temperature for 1 hr and washed again. The membranes were disposed using an electrochemiluminescence (ECL) kit (Thermo Scientific/Pierce, Rockford, IL, USA) according to the manufacturer’s protocol. The pictures were detected by a chemiluminescence detection system (Bio-Rad Laboratories, Hercules, CA, USA). The density of the immunoreactive bands was analyzed using ImageJ 1.41 (National Institutes of Health, Bethesda, MD, USA).

***Histopathological analysis***

The left kidney specimens were sectioned in blocks and fixed in 10% formalin. After fixation, tissues were dehydrated with a graded series of ethanol and xylene, embedded in paraffin, cut into 4 µm sections and stained with Mayer’s hematoxylin and eosin (H&E). The sections were observed by upright optical microscopy at 400 × magnification (NIKON ECLIPSE E100, Nikon, Japan). Tubular injury scores were analyzed by counting the percent of tubules that displayed cell necrosis, tubule dilatation, loss of brush border, and cast formation by an expert renal pathologist. And scored as: 0, none; 1, <10%; 2, 10% to 25%; 3, 25% to 75%; 4, >75%(15).

***Statistical analysis***

Data analysis and charts were generated by the GraphPad Prism 8.3.0 software package (GraphPad Software, USA). All results are expressed as the mean ± standard error of the mean (SEM). Statistical comparisons were made using Student’s *t*-test and one-way analysis of variance (ANOVA) followed by Tukey’s test. Statistically significant differences were set at *P*<0.05 or *P*<0.01.

## Results

***Effects of T-1 on CDDP-induced renal injury***

As demonstrated in Table 1, the CDDP group exhibited significant (*P*<0.01) increases in BUN and CRE levels compared with the control group. Both 10 mg/kg and 30 mg/kg treatments of T-I significantly (*P*<0.01) attenuated the levels of BUN. Moreover, treatment with 30 mg/kg T-I for 3 consecutive days significantly (*P*<0.05) decreased serum CRE levels. The levels of CRE were decreased in CDDP+ T-I (10 mg/kg) group, but the difference was not statistically significant (*P*=0.0587) as compared to the CDDP group, which might be due to huge variation of data.

Administration of CDDP significantly inhibited (*P*<0.01) anti-oxidant defense actions in kidneys, as presented by elevated levels of MDA and decreased GSH levels and CAT activities in comparison with control mice (Table 1). T-I treatment at 10 and 30 mg/kg significantly (*P*<0.05 and *P*<0.01, respectively) reduced the levels of MDA compared to the mice receiving CDDP alone. Furthermore, treatment with T-I (30 mg/kg) significantly reversed the CDDP-induced decrease in GSH and CAT levels compared with those of CDDP-treated mice (*P*<0.05 and *P*<0.01, respectively) (Table 1).

***Effects of T-1 on the expression of SOD2 protein***

**Figure 2:**
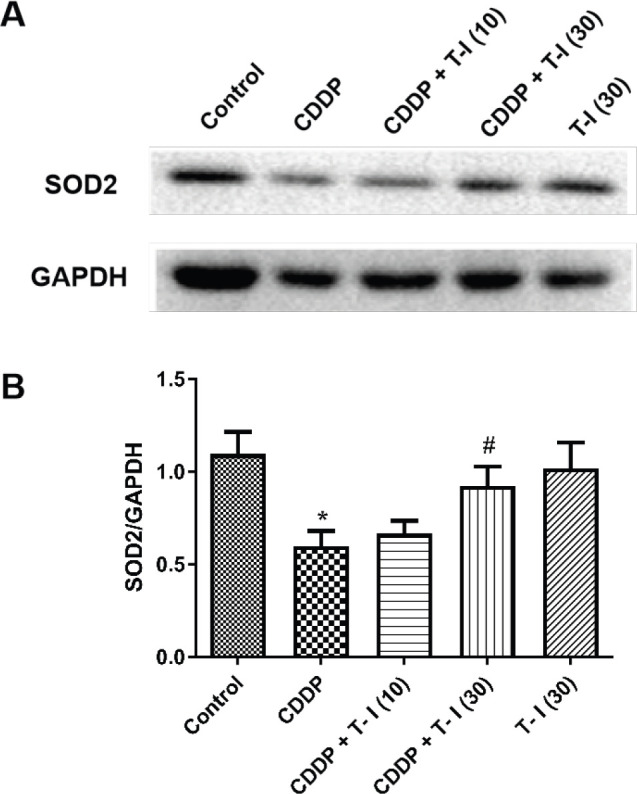
Effect of T-I on the expression of SOD2 protein with/without CDDP treatment. Representative Western blot images show CDDP- or T-I-induced SOD2 changes (A). The data are shown as the means ± SEM of four independent experiments (B). *P<0.05 compared with the control group; #*P*<0.05 compared with the CDDP group. CDDP: cisplatin; T-I: tanshinone I; SOD2: superoxide dismutase 2; GAPDH: glyceraldehyde-3-phosphate dehydrogenase

As shown in Figure 2, administration of CDDP significantly (*P*<0.05) decreased the expression levels of SOD2 protein compared to the control group. In contrast, treatment with T-I (30 mg/kg) significantly (*P*<0.05) increased the protein levels of SOD2 compared to the CDDP-administered mice.

***Effects of T-I on CDDP-mediated kidney histopathological changes***

**Figure 3:**
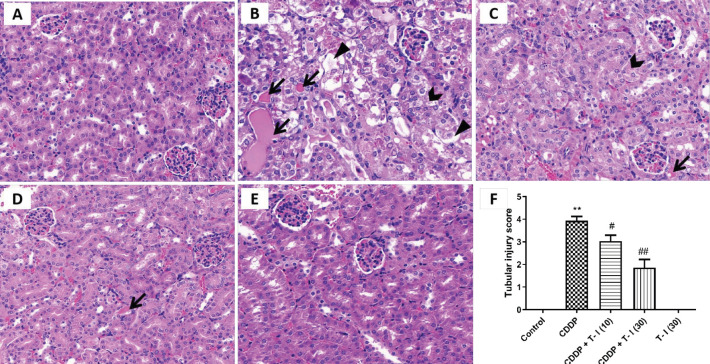
Effects of T-I on CDDP-induced kidney histopathological changes. (A): Control group; (B): CDDP group; (C): CDDP + T-I (10) group; (D): CDDP + T-I (30) group; (E): T-I (30) group; (F): tubular score. Arrows show intratubular cast formation, swallow-tail form shows granular degenerations, and triangle shows tubular epithelial cell detachment. **P< 0.01 compared with the control group; # *P*< 0.05, ##*P*< 0.01 compared with the CDDP group. (Hematoxylin and eosin staining, magnification×400). CDDP: cisplatin; T-I: tanshinone I

Both the control and T-I (30 mg/kg) groups showed completely normal renal tissues, characterized by clear tubular and glomerular structures with clear and normal nuclei (Figure 3A and 3E). The kidneys of CDDP-treated mice exhibited obvious tubular injury, including tubular dilatation, loss of brush border, granular degeneration, tubular epithelial cell detachment and intraluminal cast formation in proximal convoluted tubules (Figure 3B). However, treatment with T-I (10 and 30 mg/kg) for 3 consecutive days markedly (*P*<0.05 and *P*<0.01) improved the structural changes induced by CDDP treatment (Figure 3C and 3D).

**Table 1:** Effects of T-1 on CDDP-induced renal dysfunction and changes in renal anti-oxidants in mice

	**Control**	**CDDP**	**CDDP+ T-I (10mg/kg) **	**CDDP + T-I (30 mg/kg)**	**T-I** ** (30 mg/kg)**
**BUN** **(mmol/L)**	**8.17±0.41**	62.49±7.92**	**36.43±3.00** ^##^	**32.32±6.09** ^##^	**8.10±0.15**
**CRE** **(** **μmol/L)**	**6.25±0.48**	117.33±33.49**	**52.75±5.66**	**33.00±12.06** ^#^	**10.5±2.50**
**MDA** **(nmol/** **mg protein)**	**0.84±0.03**	1.30±0.05**	**1.01±0.07** ^#^	**0.88±0.04** ^##^	**1.00±0.05**
**GSH** **(μmol/mg protein)**	**36.47±4.06**	16.23±1.30**	**17.73±2.25**	**29.27±4.46** ^#^	**27.30±2.59**
**CAT** **(U/** **mg protein)**	**1.12±0.02**	0.46±0.04**	**0.56±0.04**	**0.78±0.03** ^##^	**1.01±0.09**

CDDP: cisplatin; T-I: tanshinone I; BUN: blood urea nitrogen; CRE: creatinine; MDA: malondialdehyde; GSH: glutathione; CAT: catalase. Values are presented as means ± SEM (n = 5). **P<0.01 compared with the control group; #*P*<0.05, ##*P*<0.01 compared with the CDDP group

## Discussion

In the present study, we investigated the effects of T-I, one of the major phenanthrenequinone compounds extracted from the Chinese herb Danshen, on CDDP-induced nephrotoxicity in mice. We demonstrated that T-I (especially 30 mg/kg, IP) attenuates CDDP-induced depletion of GSH, reduction in SOD2 protein expression and CAT activity, and an increase of MDA levels in the kidneys of mice. Treatment with this agent is associated with remarkable improvement of renal function. These results are consistent with our previous *in vitro* results (12) and further verify the protective effect of T-I against CDDP-induced nephrotoxicity *in vivo*.

CDDP is eliminated predominantly by the kidneys, and renal platinum concentration reaches to peak at 72 hr after a single intraperitoneal injection of CDDP; furthermore, BUN and CRE both increase rapidly from 48 to 120 hr (16). Based on this background, we established a mouse CDDP model of acute kidney injury (AKI) by a single IP injection of 20 mg/kg CDDP followed by euthanasia 3 days later. This method has been confirmed to be successful by some previous studies (13, 17). Similarly, in our study, treatment with CDDP resulted in obvious nephrotoxicity, as demonstrated by significant increases in serum CRE and BUN levels and morphological changes in kidney tubules.

It has been reported that oxidative stress plays an important role in CDDP-induced nephrotoxicity(4). Oxidative stress mainly occurs when the production and consumption of free radicals are unbalanced. CDDP may induce mitochondrial dysfunction and increase reactive oxygen species (ROS) production by interrupting the respiratory chain (18). Furthermore, depletion or inactivation of glutathione (GSH), superoxide dismutase (SOD), catalase (CAT) and related anti-oxidants by CDDP can induce a shift in the cellular redox status, leading to the accumulation of endogenous ROS and oxidative stress within the cells(1, 19). Thus, enhancing the cellular anti-oxidant response to counteract the increase in ROS has become a common treatment for CDDP-induced AKI. At present, several novel anti-oxidant compounds, such as sumatriptan and tangeretin, have been evaluated to protect against CDDP-induced renal injuries (5, 7).

T-I, a Danshen derivative, is confirmed to have high oxidation resistance* in vivo* and *in vitro *(9, 10, 12). Feng *et al*. found out that pretreatment with T-I at 30 mg/kg for 3 days significantly alleviated aristolochic acid I-induced kidney injury in mice (11). In another report, it was shown that T-I pretreatment at 10 mg/kg for 3 days remarkably reduced 6-hydroxydopamine-induced striatal oxidative stress and ameliorated dopaminergic neurotoxicity (14). Our previous studies confirmed that T-I attenuated CDDP-induced nephrotoxicity in HK-2 cells (12). Therefore, in this study, we selected two doses of T-I, 10 mg/kg and 30 mg/kg, to explore the protective effect of T-I on CDDP-induced nephrotoxicity. As we predicted, T-I administration to CDDP-treated mice counteracted the induced renal dysfunction and morphological abnormalities, especially at the dose of 30 mg/kg T-I. 

A previous report indicated that T-I could present potential neuroprotection against neuronal damage by increasing the production of anti-oxidants, including total anti-oxidant capacity, GSH, total SOD and CAT, and reducing the production of pro-oxidants (9). Furthermore, T-I was demonstrated to upregulate levels of mitochondrial GSH and afford mitochondrial protection against hydrogen peroxide by activating Nrf2 in SH-SY5Y cells (20). In addition, our previous* in vitro* studies confirmed that T-I pretreatment significantly reduced intracellular ROS levels and enhanced the anti-oxidant activity and survival rate of HK-2 cells after CDDP exposure by activating the Nrf2/ARE signaling pathway (12). In the present study, we further found that treatment with T-I at 30 mg/kg for 3 days significantly increased renal GSH levels and the activity of endogenous anti-oxidant enzymes, including SOD2 and CAT, in CDDP-treated mice, which demonstrated that T-I, as an anti-oxidant, could protect against CDDP-induced acute kidney injury* in vivo*. 

MDA, as one of the end products of lipid peroxidation, is constantly used as a marker of oxidative stress (1). Our results show that CDDP treatment induced renal lipid peroxidation (measured by MDA production), which was paralleled by the deterioration of renal structure and function. These results are consistent with previous reports showing that CDDP-induced lipid peroxidation is associated with altered renal function (17, 21). Of interest, the results of the current study revealed that treatment with T-I for 3 days significantly reduced the increase in MDA levels in the kidney tissues of CDDP-treated mice. Consistent with our research, recent studies have shown that T-I suppresses lipid peroxidation in H_2_O_2_-treated and methylglyoxal-treated human neuroblastoma SH-SY5Y cells by decreasing the levels of MDA (20, 22). These results supported that T-I had anti-oxidant activity* in vitro* and* in vivo*.

## Conclusion

The results of this study showed that T-I protected against CDDP-induced nephrotoxicity by reducing renal lipid peroxidation and increasing the production of endogenous anti-oxidants. T-I can be potentially used to prevent CDDP induced nephrotoxicity. However, this needs to be confirmed by measuring further biomarkers of oxidative stress and inflammation. Additionally, molecular studies should be conducted to unravel nephroprotective mechanisms.

## Authors’ Contributions

YW Study conception and design; YW, YZ, YT, JL Data processing, collection, perform experiment; YW, YZ, ZH, QH Analysis and interpretation of results; YW Draft manuscript preparation, visualization; ZH, WT, YP, YG, YH, XZ Critical revision or editing of the article; YP, ZH Supervision of the research; YW, YZ, YT, JL, ZH, WT, QH, YP, YG, YH, XZ Final approval of the version to be published. 

## Conflicts of Interest

All authors declare that there are no conflicts of interest in this study.

## References

[1] Holditch SJ, Brown CN, Lombardi AM, Nguyen KN, Edelstein CL (2019). Recent advances in models, mechanisms, biomarkers, and interventions in cisplatin-induced acute kidney injury. Int J Mol Sci.

[2] Barabas K, Milner R, Lurie D, Adin C (2008). Cisplatin: A review of toxicities and therapeutic applications. Vet Comp Oncol.

[3] Miller RP, Tadagavadi RK, Ramesh G, Reeves WB (2010). Mechanisms of cisplatin nephrotoxicity. Toxins.

[4] Manohar S, Leung N (2018). Cisplatin nephrotoxicity: A review of the literature. J Nephrol.

[5] Bazmandegan G, Amirteimoury M, Kaeidi A, Shamsizadeh A, Khademalhosseini M, Nematollahi MH (2019). Sumatriptan ameliorates renal injury induced by cisplatin in mice. Iran J Basic Med Sci.

[6] Ray PD, Huang B-W, Tsuji Y (2012). Reactive oxygen species (ROS) homeostasis and redox regulation in cellular signaling. Cell Signal.

[7] Nazari Soltan Ahmad S, Rashtchizadeh N, Argani H, Roshangar L, Ghorbanihaghjo A, Sanajou D (2019). Tangeretin protects renal tubular epithelial cells against experimental cisplatin toxicity. Iran J Basic Med Sci.

[8] Zhou S, Chen W, Su H, Zheng X (2013). Protective properties of tanshinone I against oxidative DNA damage and cytotoxicity. Food Chem Toxicol.

[9] Dai C, Liu Y, Dong Z (2017). Tanshinone I alleviates motor and cognitive impairments via suppressing oxidative stress in the neonatal rats after hypoxic-ischemic brain damage. Mol Brain.

[10] Tao S, Zheng Y, Lau A, Jaramillo MC, Chau BT, Lantz RC (2013). Tanshinone I activates the Nrf2-dependent anti-oxidant response and protects against As(III)-induced lung inflammation in vitro and in vivo. Anti-oxid Redox Signal.

[11] Feng C, Xie X, Wu M, Li C, Gao M, Liu M (2013). Tanshinone I protects mice from aristolochic acid I-induced kidney injury by induction of CYP1A. Environ Toxicol Pharmacol.

[12] Wang Y, Xi Y, Peng Y, Shen F, Deng Z, Zhang X (2018). Tanshinone I prevents cisplatin-induced cytotoxicity through Nrf2 activation in human renal proximal tubular epithelial cells. Lat Am J Pharm.

[13] Kang L, Zhao H, Chen C, Zhang X, Xu M, Duan H (2016). Sappanone A protects mice against cisplatin-induced kidney injury. Int Immunopharmacol.

[14] Jing X, Wei X, Ren M, Wang L, Zhang X, Lou H (2016). Neuroprotective effects of Tanshinone I against 6-OHDA-induced oxidative stress in cellular and mouse model of Parkinson’s disease through upregulating Nrf2. Neurochem Res.

[15] Zhang W, Hou J, Yan X, Leng J, Li R, Zhang J (2018). Platycodon grandiflorum saponins ameliorate cisplatin-induced acute nephrotoxicity through the NF-kappaB-mediated inflammation and PI3K/Akt/apoptosis signaling pathways. Nutrients.

[16] Levi J, Jacobs C, Kalman SM, McTigue M, Weiner MW (1980). Mechanism of cis-platinum nephrotoxicity: Effects of sulfhydryl groups in rat kidneys. J Pharmacol Exp Ther.

[17] Bazmandegan G, Fatemi I, Kaeidi A, Khademalhosseini M, Fathinejad A, Amirteimoury M (2021). Calcium dobesilate prevents cisplatin-induced nephrotoxicity by modulating oxidative and histopathological changes in mice. Naunyn Schmiedebergs Arch Pharmacol.

[18] Chirino YI, Pedraza-Chaverri J (2009). Role of oxidative and nitrosative stress in cisplatin-induced nephrotoxicity. Exp Toxicol Pathol.

[19] Pabla N, Dong Z (2008). Cisplatin nephrotoxicity: mechanisms and renoprotective strategies. Kidney Int.

[20] de Oliveira MR, Furstenau CR, de Souza ICC, da Costa Ferreira G (2017). Tanshinone I attenuates the effects of a challenge with H2O2 on the functions of tricarboxylic acid cycle and respiratory chain in SH-SY5Y cells. Mol Neurobiol.

[21] Ismail RS, El-Awady MS, Hassan MH (2020). Pantoprazole abrogated cisplatin-induced nephrotoxicity in mice via suppression of inflammation, apoptosis, and oxidative stress. Naunyn Schmiedebergs Arch Pharmacol.

[22] Furstenau CR, de Souza ICC, de Oliveira MR (2019). Tanshinone I induces mitochondrial protection by a mechanism involving the Nrf2/GSH axis in the human neuroblastoma SH-SY5Y cells exposed to methylglyoxal. Neurotox Res.

